# 

**Published:** 1914-05-23

**Authors:** 


### CONTENTS.

| | PAGE |
|---|---|
| **St. George's and the Westminster Hospitals** | 197 |
| **The Future Relations of Medicine and the State** ... ... ... ... ... ... ... | 198 |
| **Hospital and Institutional News** ... ... ... | 199 |

Our Prize Scheme for Equipment "Tips"—Director of Research under the Insurance Act—A Poor-Law Medical Officer and the Use of Brandy—The Isolation Hospital "Gardener"—Male Nurses in Poor-Law Infirmaries—The Sex Question in Infirmary Administration—A Nursing Training School for Men—Is the Future to Nursing by Men?—A Provincial Hospital Garden Party—A Medical Mayor on Music—The River Ambulance Service—The Continuous-immersion Bath—The Marriage of Faith and Science—Mental Hospitals and Suggestion—The Milk Problem in Military Hospitals—Sudden Illness of a London Hospital Chairman—Mr. A. J. Balfour and Alton Hospital—A Practical Tribute to a Hospital Staff—Typhoid and the Mexican Campaign—What the South London Hospital Offers to Women Doctors—Workhouse Wages for the Physically Unfit—Death of an ex-Hospital Almoner—Rubber Flooring for Guy's Hospital—Personal Service in Bristol—Panel Chemists and the Drug Funds—The Growth of the Pharmaceutical Society—Latest Enlargement of Poor-Law Areas—Two Serious Omissions—The Earthquake and the Drug Market.

| | PAGE |
|---|---|
| **The Surgical Congress: July 27–31** ... ... | 205 |
| Institutional Personalities. | |
| **Modern Methods Series** ... ... ... ... | 206 |
| Treatment of Hay-Fever—Emetine in Tuberculosis. | |
| **The Orthopædic Department** ... ... ... | 207 |
| VII. The Plaster Room (*continued*). | |
| **Recurring Jaundice in Successive Pregnancies** ... ... ... ... ... ... ... | 208 |
| **Reports on Hospitals of the United Kingdom**— | 209 |
| By SIR HENRY BURDETT, K.C.B., K.C.V.O. | |
| Croydon General Hospital. | |
| **Buildings Contemplated** ... ... ... ... | 210 |

[*See over.*]

### CONTENTS—(*continued*).

PAGE

**National Insurance Act Developments** ... ... 211
Forthcoming Developments Indicated in the Budget.

**Gifts and Bequests** ... ... ... ... ... 212

**The Editor's Letter-Box** ... ... ... ... 213
The Uckfield Cottage Hospital—What Keeps a Man off the Panel?—The Abuse of Tuberculin—Human Hibernation.

**Round the World** ... ... ... ... ... 215

Fellow-Workers and their Doings.

**Legal Notes for Institutional Workers** ... 216

The Wage-earning Value of the One-eyed Man.

**A Deportment Coronet** ... ... ... ... 216

**The Secret of Institutional Discipline** ... ... 217

**The Asylum Workers' Association** ... ... 218

Speeches at the Annual Meeting.

PAGE

**Two Bed-Lifters Described** ... ... ... 219

**Medical Appointments** ... ... ... ... 219

**The Staff at Gravesend Hospital** ... ... 220

**The Book Market** ... ... ... ... ... 220

**Some Historic Children's Hospital Cots** ... 221
The Royal, the Humorous, the Heroic.

**The National Medical Union (Official)** ... ... 222
Medical Forethought in State Medicine.

**The Institutional Worker** ... ... (Suppl.) 1
Baths for Continuous Immersion.
Questions for May.
Questions Invited.
The Sick and in Need.
Miscellaneous.
Employment and Training.
Acknowledgments.

